# Understanding and Remediating Social-Cognitive Dysfunctions in Patients with Serious Mental Illness Using Relational Frame Theory

**DOI:** 10.3389/fpsyg.2016.00143

**Published:** 2016-02-12

**Authors:** Annemieke L. Hendriks, Yvonne Barnes-Holmes, Ciara McEnteggart, Hubert R. A. De Mey, Gwenny T. L. Janssen, Jos I. M. Egger

**Affiliations:** ^1^Centre of Excellence for Neuropsychiatry, Vincent van Gogh Institute for PsychiatryVenray, Netherlands; ^2^Behavioural Science Institute, Radboud UniversityNijmegen, Netherlands; ^3^Donders Institute for Brain, Cognition and Behaviour, Radboud UniversityNijmegen, Netherlands; ^4^Department of Experimental-Clinical and Health Psychology, Ghent UniversityGhent, Belgium

**Keywords:** schizophrenia, autism, social cognition, perspective-taking, Relational Frame Theory

## Abstract

Impairments in social cognition and perspective-taking play an important role in the psychopathology and social functioning of individuals with social anxiety, autism, or schizophrenia-spectrum disorders, among other clinical presentations. Perspective-taking has mostly been studied using the concept of Theory of Mind (ToM), which describes the sequential development of these skills in young children, as well as clinical populations experiencing perspective-taking difficulties. Several studies mention positive results of ToM based training programs; however, the precise processes involved in the achievement of these improvements are difficult to determine. Relational Frame Theory (RFT) is a modern behavioral account of complex cognitive functions, and is argued to provide a more precise approach to the assessment and training of perspective-taking, among other relational skills. Results of RFT-based studies of perspective-taking in developmental and clinical settings are discussed. The development of training methods targeting perspective-taking deficits from an RFT point of view appears to provide promising applications for the enhancement of current treatments of people with social-cognitive dysfunctions.

## Social Cognition

Social cognition has long been a focus in social, cognitive, and clinical psychology. Its importance is not surprising given that social cognition allows us to select, interpret and use social information (Aronson and Wilson, 2005), and that these in turn are critical to our understanding of self and others, the wider social context and how we can relate to these. It is equally well established that perspective-taking skills are central to social cognition, given that they enable us to distinguish between mental states of self and others, and to take the perspective of another. Indeed, perspective-taking is a pre-requisite for many aspects of complex social interaction, including empathy, irony, humor, and deception (Heagle and Rehfeldt, 2006; Barnes-Holmes, unpublished doctoral thesis).

Given the centrality of perspective-taking in social cognition, individuals with problems in the former naturally display a broad range of problems in the latter (Brüne and Brüne-Cohrs, 2006; Couture et al., 2006). For example, individuals with a diagnosis of autism spectrum disorder (ASD) are well-known for impairments in mental state attribution, social reciprocity, emotion perception and empathy, all of which negatively affect social interaction and communication (Yirmiya et al., 1996; American Psychiatric Association [APA], 2000; Baron-Cohen et al., 2000).

It was suggested by Frith (1992) that similar deficits occur in individuals with a diagnosis of schizophrenia and account, to some extent, for characteristic features of social functioning that are observed in both positive and negative symptoms. Specifically, impairments in discriminating the mental states of the self and others may contribute to hallucinations and delusions, as well as the absence of meaningful social interaction. Furthermore, Hardy-Bayle (1994) argued that deficits in mental state attribution are associated with thought disorganization and the inability to abstract contextual information regarding social behavior. At the present time, there is a well-supported body of evidence from social-cognitive studies to suggest that individuals with a diagnosis of schizophrenia show impairments in mental state attribution (Corcoran et al., 1995; Sprong et al., 2007) and perspective-taking (Langdon et al., 2001; Villatte et al., 2010b), even during phases of remission (Bora et al., 2009).

During acute psychotic phases, treatment of schizophrenia mainly targets hallucinations and delusions through pharmacological means, with some short-term success (Slooff et al., 2006). However, life-long medication is often recommended (Kissling, 1991; Davis, 2006) and significant concerns with this type of regime surround the many unpleasant side effects observed (e.g., extrapyramidal symptoms, hypotension and intestinal complaints) and problems with medication compliance (Slooff et al., 2006). Some of these side effects have been reduced with the development of second-generation antipsychotic drugs, and improvements in negative symptoms have been reported (Woo et al., 2009). Still, pharmacological regimes that focus primarily on the suppression of positive symptoms leave impairments in social functioning and perspective-taking untouched. Psychosocial treatment focusing on these factors, therefore, complements current treatments of schizophrenia (Kern et al., 2009; Woo et al., 2009).

Given the pivotal role of perspective-taking in social functioning, it is not surprising that many other clinical categories that include social impairments also present with difficulties in perspective-taking or mental state attribution. Results from social-cognitive studies indicate that these include schizotypy (Langdon and Coltheart, 1999; Langdon et al., 2001; Platek et al., 2003; Pickup, 2006), frontotemporal dementia (Gregory et al., 2002), brain damage/degenerative brain disease (for an overview see Brüne, 2005), alcohol use disorder (Uekermann and Daum, 2008), cocaine use disorder (Hulka et al., 2013, 2015; Preller et al., 2014a,b), borderline personality disorder (Herpertz and Bertsch, 2014; Sharp et al., 2015), depression (Lee et al., 2005; Wang et al., 2008; Ladegaard et al., 2014), anxiety (Bodner et al., 2012; Samson et al., 2012) and obsessive compulsive disorder (Sayin et al., 2010). As a result, perspective-taking skills are of pivotal importance to many domains in clinical research and practice.

## Perspective-Taking

The dominant theoretical paradigm in the study of perspective-taking is Theory of Mind (ToM). Originally described by Premack and Woodruff (1978), ToM refers to the ability to infer the beliefs, intentions, thoughts and emotions of the self and others, and advocates of the theory have described the natural trajectory of these skills from simple to complex perspective-taking (Howlin et al., 1999). In typical development, perspective-taking skills are believed to be fully developed by around age six, although some evidence suggests that these repertoires remain somewhat inflexible even until late adolescence or early adulthood (Keysar et al., 2003; Dumontheil et al., 2010).

Many theoretical accounts have been proposed within ToM research (Ensink and Mayes, 2010). As the most prevalent account of ToM, Premack and Woodruff (1978) proposed that ToM can be viewed as a theory that children develop about others’ minds, also referred to as the theory-theory account (Premack and Woodruff, 1978; Astington and Gopnik, 1991). Simulation theorists, on the other hand, argue that people identify with another person’s perspective and are, thereby, able to simulate his/her experience (Harris et al., 1989). Proponents of nativistic accounts state that ToM is a result of innate processes in the brain, and that the development of these skills is dependent on the maturation of these mechanisms (for an overview see Ensink and Mayes, 2010). Indeed, results from neuroimaging studies indicate that specific areas of the brain (temporal lobes, inferior parietal cortex, frontal lobes) may be involved in ToM (Brüne and Brüne-Cohrs, 2006). Evidence also suggests that “mirror neurons” play a part in the development of these skills (Mayes et al., 2009). This specific type of neuron is activated both during an action and during observation of that same action in another person, and is therefore argued to be of importance in imitation and understanding of intentions (Ensink and Mayes, 2010). Furthermore, ToM skills have been linked to executive functioning (e.g., Frye et al., 1995; Perner and Lang, 2000).

As a mainstream developmental approach to perspective-taking, ToM has substantial descriptive and conceptual merit, and was the first to offer a coherent account of the putative components of these pivotal skills. In addition, evidence supports the application of these concepts through remedial interventions, either aimed at ToM specifically or as a part of broader training in social cognition. For example, Fisher and Happé (2005) reported improvements in ToM performance in children diagnosed with ASD, while Gevers et al. (2006) described similar outcomes with children with a pervasive developmental disorder. ToM skills have also been successfully trained in adults with high-functioning autism (Turner-Brown et al., 2008). Positive results for social cognitive ToM training were found as well in patients with schizophrenia; for an extensive overview see Horan et al. (2008) or Brüne and Juckel (2010). Although these positive clinical outcomes are promising, it is difficult to determine precisely the processes of perspective-taking that are being enhanced during training. That is, the middle-level and higher-level concepts employed in ToM (e.g., mental state attribution and social cognition) do not directly specify psychological or behavioral processes. So while one might realistically assume, as the evidence suggests, that perspective-taking and social cognition more broadly operate through similar processes or mechanisms, neither concept “explains” what those mechanisms are. This conceptual shortfall not only limits our understanding of these skills in normative development and that which underpins clinical presentations, but also restricts the development of research methodologies and interventions. In the section below, the paper explores an alternative approach to understanding perspective-taking that emanates directly from Relational Frame Theory (RFT), a modern behavioral account of human language and cognition (Hayes et al., 2001).

## Relational Frame Theory

### Key Concepts

As one would expect from a behavioral theory, RFT concerns itself with the interactions between an organism and the environment, and the account is in this respect also philosophically contextual and functional. In short, RFT proposes that language and cognition primarily involve a learning process known as *arbitrarily applicable relational responding* (AARR). In short, complex and subtle contextual cues in the environment facilitate the derivation of arbitrary relations among stimuli or events. For example, if a speaker says “apples are like oranges,” the phrase “is like” acts as a contextual cue for the listener to derive a relation of similarity, or co-ordination, between apples and oranges. In addition, the words “apples” and “oranges” themselves are co-ordinated with the actual fruits symbolized by the words. Cognitive development is believed to involve the emergence of elaborate and complex networks of these relations (Blackledge, 2003), which we use in complex cognitive functions like problem-solving, humor, and perspective-taking. For example, understanding metaphor is believed to involve relating relational networks to other relational networks (Foody et al., 2014).

Empirical evidence on RFT across several decades supports the theory’s core concept of arbitrarily applicable relational responding (i.e. deriving relations) and indicates that these complex skills proliferate in the absence of direct training (Hayes et al., 2001). Furthermore, studies have identified a number of families of relational frames (i.e., patterns of relating) that share the same core processes but have different formal features. These may be summarized as: co-ordination (i.e., similarity or sameness); distinction (difference); opposition; comparison; hierarchy; and perspective-taking (known in RFT as deictic relations). There is also a growing body of evidence on the relationship between these skills and: language/higher cognition; IQ; developmental delays; and other clinical presentations (e.g., schizophrenia and social anxiety). For a recent book-length review of this evidence, see Dymond and Roche (2013).

### The RFT Account of Perspective-Taking

For RFT, the deictic or perspective-taking relations lie at the very heart of language, complex cognition and a sense of self, because they comprise the constant perspective from which a verbally sophisticated individual interprets his/her environment. Specifically, the theory proposes three types of perspective-taking relations: the interpersonal relations of I and YOU; the spatial relations of HERE and THERE; and the temporal relations of NOW and THEN. Indeed, these concepts are highly similar to those widely used in the developmental literature and the specific literature on the concept of self (Howlin et al., 1999; Barnes-Holmes et al., 2001).

According to RFT, we learn to derive deictic relations across everyday questions such as “What are you doing there?” and “What was it like yesterday?” That is, common social interactions create a history in which specific words come to function as contextual cues for deriving the perspective-taking relations in a given context. Furthermore, the interpersonal, spatial and temporal relations interact with each other in various ways, thereby adding both complexity and constancy to our perspectives. For example, I always operates from the HERE and NOW, and from that perspective YOU/OTHERS are always anchored THERE and THEN. For RFT, these behaviors of relating comprise core features of one’s sense of self, as well as one’s sense of others.

Early empirical investigations of the perspective-taking relations, in RFT work with young children, have explored whether the three types of relations are in fact functionally distinct from one another, hence each constituting a valid functional concept. With this purpose, Barnes-Holmes (unpublished doctoral thesis) developed an extensive protocol exploring the three relations and how they interact with one another at different levels (see **Table 1** for a detailed overview of the trial types). For example, in a simple I-YOU trial, a child was asked to imagine a scenario in which “I have a green brick and you have a red brick” and was then asked “Which brick do I/you have?” This trial simply gages whether the child can respond correctly on the basis of I versus YOU (i.e., whether the words “I” and “you” control the appropriate perspectives). Simple HERE–THERE trials follow a similar hypothetical format: “I am sitting here on the blue chair and you are sitting there on the black chair. Where am I sitting? Where are you sitting?” And the research also investigated simple NOW–THEN relations. However, in order to gage whether the child can respond correctly on the basis of NOW versus THEN, the early research parsed out I and YOU trials to circumvent problems in derivation. For example, if I say “Yesterday I was reading, today you are watching TV,” you will not have enough information to derive either what I am doing today or what you were doing yesterday. This problem only applies to NOW–THEN relations. However, it is important to note that neither spatial nor temporal relations have meaning without an interpersonal perspective from which to determine them (i.e., there is no “here” or “now” without I).

**Table 1:** The core relational skills involved in perspective-taking (McHugh et al., 2008).

Relation type	Level of relational complexity		
	Simple relations	Reversed relations	Double reversed relations
I–YOU	Simple I–YOU	Reversed I–YOU	
HERE–THERE	Simple I–YOU and simple HERE–THERE	Reversed I–YOU and simple HERE–THERE Simple I–YOU and reversed HERE–THERE	Double reversed I–YOU and HERE–THERE
NOW—THEN	I and simple NOW–THEN YOU and simple NOW–THEN	I and reversed NOW–THEN YOU and reversed NOW–THEN	
	I and simple HERE–THERE and simple NOW–THEN	I and reversed HERE-THERE and simple NOW–THEN I and simple HERE–THERE and reversed NOW–THEN	I and double reversed HERE–THERE and NOW–THEN
	YOU and simple HERE–THERE and simple NOW–THEN	YOU and reversed HERE–THERE and simple NOW–THEN YOU and simple HERE–THERE and reversed NOW–THEN	YOU and double reversed HERE–THERE and NOW–THEN

While these three types of deictic relations already show considerable complexity, particularly in the ways in which they interact with each other, Barnes-Holmes (unpublished doctoral thesis) demonstrated even greater complexity by reversing and further integrating the three relations. In short, trials were created to assess the extent to which children were capable of taking the perspective of another (e.g., reversing the functions of “I” and “you”). Being able to do this would likely indicate some level of relational flexibility. Indeed, complex social interactions are embedded within the need to see the world as someone else sees it, without losing our own perspective. Other variations in the protocol include reversed HERE–THERE and NOW–THEN relations, as well as two *simultaneously reversed* deictic relations, thus increasing complexity and interaction between relations even further. While these types of complex relational tasks would be rarely required in natural language, there may be instances, for example in complex logic, where one would need the flexibility to be able to separate the relations in this way.

The findings from the original research, and much subsequently, have indicated clear functional distinctions among the three perspective-taking relations and among the three levels of relational complexity (simple, reversed and double reversed). For example, participants of almost all ages show competence on I–YOU relations, poorer performances on HERE–THERE relations, and weaker still performances on NOW–THEN relations (McHugh et al., 2004). Furthermore, they show some competence on simple relations, poorer performances on reversed relations and even poorer performances on double reversals. The evidence also suggests a developmental trend in the emergence of perspective-taking skills according to these sequences, which is in line with the developmental literature more broadly (Howlin et al., 1999). Similarly, correlational analyses suggest at least some potential overlap between the processes underpinning both relational perspective-taking and ToM (Villatte et al., 2008, 2010b).

### The Role of Deictic Relations in Clinical Samples

Following the early developmental RFT studies on perspective-taking, the Barnes-Holmes protocol has been administered in several clinical samples known for their perspective-taking difficulties and interesting patterns of results have been observed. For example, children with ASD have been found to have lower accuracies than their typically developing peers, especially regarding deictic reversals (Rehfeldt et al., 2007). McGuinness (unpublished master’s thesis) administered the protocol to nine participants diagnosed with Asperger’s syndrome, and found diminished performances on both reversed and double-reversed trials. Furthermore, Villatte et al. (2010b) found lower accuracies on the deictic protocol with individuals with a diagnosis of schizophrenia, when compared to a control group. Again, these difficulties were mainly recorded in the reversal trials (Villatte et al., 2010b). Evidence of impaired flexibility in deictic responding has also been recorded in participants categorized as high in social anhedonia, in this case on double reversal trials (Villatte et al., 2008). Also, lower accuracies on reversal trials have been found with individuals experiencing social anxiety (Janssen et al., 2014). Difficulties in responding to deictic relations may result in problems in other areas of social cognition as well. For example, in a similar relational protocol, Villatte et al. (2010a) found that people with schizophrenia or high social anhedonia were less accurate in attributing true or false beliefs to another. Vilardaga (2009) also argued that empathy could be understood by examining deictic perspective-taking relations. Similar to more cognitive accounts of empathy (Lamm et al., 2007), he emphasizes the importance of being able to define the origin of the expected feelings, or in RFT terms, of being able to accurately distinguish between one’s own perspective (HERE-NOW) and what is spoken about (THERE-THEN).

Taken together, it is interesting that the relational protocol has shown precise deficits in perspective-taking in samples, whose diagnoses are characterized by difficult social relationships. Furthermore, the combined results of these studies (based on visual inspection of the data) show that these clinical samples generally perform best on I–YOU trials, followed by NOW–THEN trials, with poorer performances on HERE–THERE trials. That is, while these individuals appear to experience more difficulties in complex perspective-taking, the data also indicates possible differences in the development of perspective-taking relations compared to non-clinical populations. These results are consistent with the Hardy-Baylé model of schizophrenia as well, as they indicate that processing of spatial–temporal contextual information in social interaction is deficient in people with schizophrenia-spectrum disorders (Hardy-Bayle, 1994; Sarfati and Hardy-Baylé, 1999).

### Remediating Deficits in Deictic Relations

Currently, one area of RFT research is focusing on the remediation of these deficits as observed in various clinical samples. Specifically, several studies with typically developing children have already highlighted the utility of combining the original protocol trials with feedback as a successful means of establishing the target relational performances. For example, McHugh et al. (2008) demonstrated how these perspective-taking skills were successfully facilitated or established in small samples of young children (Barnes-Holmes, unpublished doctoral thesis; McHugh, unpublished doctoral thesis). Similar studies have found that these effects also generalize to natural language (Heagle and Rehfeldt, 2006; Weil et al., 2011). Overall, these results indicate that deictic relations can be successfully trained when they are deficient, and results from a few recent studies suggest that the same principles apply to clinical populations experiencing perspective-taking difficulties. In one of these studies, Jackson et al. (2014) successfully trained deictic relational responding in three children with an ASD, although generalization to ToM performance was not found (Jackson et al., 2014). The authors of that work suggested that a more extensive training protocol, targeting each perspective-taking relation separately in each level of complexity, might benefit learning outcomes with this population and help to improve generalization to ToM skills. Improvements in relational perspective-taking were also found in a study by Lovett and Rehfeldt (2014), who trained three adolescents with Asperger’s syndrome. O’Neill and Weil (2014) studied the effects of a similar training program in three patients diagnosed with schizophrenia and found improvements in deictic responding. Furthermore, all participants showed some improvements in ToM performance (O’Neill and Weil, 2014). In another study, the current authors are examining the effectiveness of relational perspective-taking training in samples with social anxiety, with initial unpublished results showing increased performances on these types of trials. In short, existing results suggest that perspective-taking can be successfully trained in clinical samples, and may thus potentially provide promising applications to benefit current treatments.

## Conclusion

Thus far, RFT research has been able to determine precisely and functionally the components that comprise perspective-taking; has allowed for a detailed developmental profile of these skills from early childhood until adulthood; and has identified several populations experiencing difficulties in this area. Results of some initial RFT studies have suggested that perspective-taking can be trained and generalized successfully by combining deictic relational trials with feedback, and these principles are currently being examined in clinical populations experiencing perspective-taking difficulties. This type of research not only helps to clarify the key role of perspective-taking in social cognitive functioning, but also provides practical methods of relational training to complement current treatments of (social cognitive) psychopathology.

## Author Contributions

Conceived and designed the paper: AH, JE, YB-H, and GJ. Analysed and interpreted the literature: AH, YB-H, and CM. Drafted the manuscript: AH. Contributed significantly to the content of the manuscript: JE, YB-H, GJ, CM, and HDM. Critically revised the manuscript JE, YB-H, GJ, CM, and HDM. Final writing and full approval of the manuscript: All authors.

## Conflict of Interest Statement

The authors declare that the research was conducted in the absence of any commercial or financial relationships that could be construed as a potential conflict of interest.
